# Phosphatases of regenerating liver downregulate PTEN to promote tumorigenesis

**DOI:** 10.1042/BST20250538

**Published:** 2026-06-25

**Authors:** Ahmed M. Abou-Shanab, Jingmei Yu, Yunpeng Bai, Zhong-Yin Zhang

**Affiliations:** 1 Borch Department of Medicinal Chemistry and Molecular Pharmacology, Purdue University, 720 Clinic Drive, West Lafayette, IN 47907, U.S.A.; 2 Institute for Cancer Research, Purdue University, 720 Clinic Drive, West Lafayette, IN 47907, U.S.A.; 3 James Tarpo Jr. and Margaret Tarpo Department of Chemistry, Purdue University, 720 Clinic Drive, West Lafayette, IN 47907, U.S.A.; 4 Institute for Drug Discovery, Purdue University, 720 Clinic Drive, West Lafayette, IN 47907, U.S.A.

**Keywords:** AKT, phosphoinositide 3-kinase, PRLs, PTEN, tumorigenesis

## Abstract

The phosphatase and tensin homolog deleted on chromosome 10 (PTEN) is one of the most frequently inactivated tumor suppressors in human cancers, serving as a critical negative regulator of phosphatidylinositol 3-kinase (PI3K)–AKT signaling. Although genetic mutation or deletion commonly underlie functional PTEN loss, accumulating evidence indicates that post-transcriptional and post-translational mechanisms also substantially contribute to PTEN suppression. Phosphatases of regenerating liver (PRLs), comprising PRL1, PRL2, and PRL3, are oncogenic phosphatases frequently overexpressed in both solid and hematological malignancies. Emerging studies reveal that PRLs can downregulate PTEN through a post-translational mechanism by direct dephosphorylation of PTEN at Tyr336, therefore promoting PTEN ubiquitination and proteasomal degradation. PRLs can also reduce PTEN expression through a post-transcriptional mechanism by dephosphorylating the inhibitory Tyr570 in JAK2, thereby activating the JAK2/STAT3-mediated miR-21 expression. These coordinated actions collectively amplify PI3K–AKT signaling, consequently promoting proliferation, survival, and metastasis. In the present review, we synthesize current knowledge of PRL structure, evolution, and functional diversity, evaluate genetic, biochemical, and organismal evidence linking PRLs to PTEN regulation, and discuss insights on PRL oncogenicity derived from experimental models. We further examine context-dependent functions of PRLs, unresolved questions regarding catalytic versus scaffold activities, and the therapeutic potential of targeting the PRL–PTEN axis. Understanding how PRLs modulate PTEN activity may reveal new strategies to restore tumor suppressor function in PTEN-deficient cancers.

## Introduction

Protein tyrosine phosphorylation is a fundamental post-translational regulatory mechanism governing signaling pathways that control numerous cellular processes [1,2]. Cellular homeostasis depends on the tightly coordinated and reversible actions of protein tyrosine kinases (PTKs) and protein tyrosine phosphatases (PTPs), which together maintain appropriate protein tyrosine phosphorylation levels and dynamics [3]. The human PTP superfamily comprises over 100 members, and dysregulation of PTP activity has been implicated in a wide range of pathologies, including cancer, metabolic disorders, and autoimmune diseases [4,5]. Within this superfamily, phosphatases of regenerating liver (PRLs), PRL1, PRL2, and PRL3, encoded by *PTP4A1, PTP4A2*, and *PTP4A3*, form a highly conserved subfamily sharing more than 75% amino acid sequence identity [6,7]. By opposing the actions of PTKs, PTPs are often regarded as negative regulators of cell signaling and are thus long thought to be products of tumor suppressor genes. Recent findings, however, have overturned this narrative, firmly establishing many PTPs, including PRLs, PTP1B, and SHP2, as oncogenic drivers [8–10]. Interestingly, PRL expressions are upregulated in numerous cancers, and increased PRL expressions are strongly correlated with late-stage metastasis and poor clinical outcomes [11–15]. These findings implicate PRLs as potential molecular markers and therapeutic targets for metastatic cancers. Aberrant overexpression of PRLs has been associated with enhanced cell proliferation and invasion through activation of multiple oncogenic signaling pathways, including the Rho family GTPases, SRC, STAT3, ERK1/2, and phosphatidylinositol 3-kinase (PI3K)/AKT cascades [10,16,17]. In addition to their effects on cellular signaling, PRLs can regulate intracellular magnesium concentrations through interactions with magnesium transporters of the cyclin M (CNNM) magnesium transporters [18–22]. Despite these extensive studies, a unifying biochemical mechanism explaining how PRLs amplify oncogenic signaling has remained elusive.

A major barrier to understanding PRL’s oncogenic propensity has been the scarcity of identified PRL substrates, thereby limiting our ability to define the signaling pathways through which PRLs exert their oncogenic effects. Moreover, the relatively weak phosphatase activity of PRLs toward artificial small molecule substrates has fueled an ongoing debate regarding whether PRLs function primarily as catalytic phosphatases or as non-catalytic scaffold proteins. Evidence supporting their non-catalytic roles include the evolutionarily conserved PRL–CNNM interaction, which regulates magnesium homeostasis and influences cellular proliferation and migration [23,24]. Conversely, the preservation of the canonical catalytic motif, formation of a phosphocysteine intermediate, characteristic burst kinetics, and emerging reports of substrate-dependent signaling regulation support a biologically relevant catalytic function [22,23,25,26]. As biological catalysts that speed up chemical reactions, enzymes display drastically different rate constants. Thus, just because PRLs exhibit poor *in vitro* activity against artificial substrates does not preclude them from functioning as genuine phosphatases *in vivo*. Indeed, conclusions drawn from catalytically impaired mutants remain difficult to interpret because many studies rely on overexpression systems while endogenous PRLs remain present, potentially masking catalytic or scaffolding contributions.

Importantly, the absence of clearly defined physiological substrates should not be interpreted as evidence against catalytic activity *per se*, but rather as a reflection of the current limitations in capturing PRL enzymatic function within its native cellular environment. Rather than arguing for mutually exclusive models, current evidence increasingly suggests that PRLs may have both catalytic and non-catalytic functions. The relative contribution of each mechanism likely depends on cellular context, subcellular localization, interacting partners, and substrate availability. In addition, analogous paradigms in phosphatase biology, such as phosphatase and tensin homolog deleted on chromosome 10 (PTEN), demonstrate that weak activity toward artificial substrates can obscure robust physiological function that becomes apparent only in the presence of native lipid or protein substrates within the appropriate subcellular environment [27,28].

Notwithstanding the above discussion, a growing body of evidence now suggests that PRLs function as upstream modulators that potentiate kinase pathways by dismantling negative regulatory checkpoints. In particular, several recent studies have identified PTEN, a central tumor suppressor and key antagonist of PI3K/AKT signaling [29], as a recurrent target for PRL-mediated downregulation [30]. Because PTEN functions in a dose-dependent manner, elucidating the regulatory mechanisms governing PTEN abundance is critical for understanding its diverse roles in tumor initiation, progression, and therapeutic resistance. Herein, we review the role of PRLs in cancer initiation and progression, focusing on their ability to amplify PI3K/AKT signaling cascades by downregulating PTEN through both post-transcriptional and post-translational mechanisms.

## PRLs as culprits of tumorigenesis

The founding member of the PRL family, PRL1, was originally identified as an immediate-early gene induced during liver regeneration, therefore giving rise to the designation ‘phosphatase of regenerating liver’ [31,32]. This discovery was subsequently followed by the identification of two closely related homologs, PRL2 and PRL3 [33]. Initial enthusiasm surrounding the oncogenic relevance of PRLs was largely driven by the observation that PRL3 is markedly overexpressed in metastatic colon cancer compared with primary tumors and normal colonic epithelium [14,34,35]. Since then, numerous studies have reported elevated PRL3 expression across a broad spectrum of malignancies, frequently correlating with advanced disease stage, enhanced metastatic potential, angiogenesis, and poor patient survival (reviewed in [34,36]). These associations have positioned PRL3 as a candidate biomarker of tumor aggressiveness and a putative therapeutic target, although the causal relationship between PRL3 expression and tumor progression remains incompletely resolved. In addition to PRL3, the expression of PRL1 and PRL2 is also found to be highly elevated in numerous cancers [7,12,37,38]. Together, these findings indicate that oncogenicity is not unique to PRL3 but is a general property of the PRL family, including PRL1 and PRL2.

Structurally, PRLs are classified within the dual-specificity phosphatase subgroup of the PTP superfamily based on their ability to dephosphorylate phosphotyrosine, phosphoserine, and phosphothreonine residues [36,39]. *In vitro* studies suggest that PRLs can also dephosphorylate phosphoinositides, although the physiological relevance of this activity continues to be an active area of inquiry [40,41]. PRLs are small (∼20 kDa) enzymes composed of a central catalytic PTP domain and a distinctive C-terminal tail harboring a polybasic region and a CAAX prenylation motif that is unique among PTPs [39,42]. Farnesylation of this CAAX motif is essential for PRL membrane association and is required, together with intact catalytic activity, for many reported PRL-dependent cellular phenotypes [43–46].

PRLs have been reported to promote oncogenic signaling by suppressing multiple negative regulatory components. PRL3 reduces the expression of C-terminal SRC kinase (CSK), thereby relieving the inhibitory tyrosine phosphorylation in SRC and enhancing SRC signaling [47]. PRLs also downregulate PTEN, a key antagonist of PI3K/AKT signaling, thereby sustaining AKT activation [48,49]. In addition, PRL3 activates EGFR signaling in cancer cells through transcriptional repression of PTP1B, an inhibitory EGFR phosphatase [50]. Although these effects converge on SRC, PI3K/AKT, and EGFR pathway activation, the underlying mechanisms differ across PRL family members. PRL3 suppresses CSK via impaired eIF2α mediated protein translation [51]. PRL2 downregulates PTEN through both post-transcriptional [17] and post-translational levels [30]. PRL3 represses PTP1B at the transcriptional level [50].

At the cellular level, altered PRL expression, particularly of PRL3, has been linked to a wide array of phenotypes such as cytoskeletal remodeling, cell–cell and cell–matrix interactions, epithelial-to-mesenchymal transition, and cell cycle regulation [52–54]. While these observations collectively suggest pleiotropic roles for PRLs in cancer biology, they have not converged on a unified signaling framework. Numerous candidate PRL-interacting proteins and substrates have been reported (Table 1). Recently, we have identified and characterized several protein substrates of PRLs, including PTEN Tyr336 [30], VCP Tyr805 [55], CBL Tyr371 [56,57], JAK2 Tyr570 [17], and p53 Ser 392 [58]. However, much of these biochemical insights have been derived from studies of PRL overexpression or depletion in diverse cell line models, raising concerns about context dependency and biological relevance. Consequently, despite more than two decades of intensive investigation, the physiological functions of PRLs in normal tissues and the molecular basis by which they drive malignant transformation remain poorly defined, which represent major unresolved challenges in the field.

**Table 1:** PRLs interacting partners and substrates and their biological functions

	Target	Biological role	Reference
**PRL1**	Cyclin M-2 (CNNM2)	Magnesium homeostasis	[59]
	ATF-7	A bZIP protein that binds specifically to CRE elements	[60]
	α-tubulin	Cellular mitosis	[61]
	p115 RhoGAP	Activates RhoA signaling by inhibiting the catalytic activity of p115 RhoGAP to drive cell motility	[16]
	Pyruvate kinase isoenzyme M2 (PKM2)	A rate-limiting enzyme in glycolysis to promote pyruvate production	[62]
	Aconitase 2 (ACO2)	Mitochondrial enzyme that catalyzes the conversion of citrate to isocitrate during the tricarboxylic acid cycle	[62]
	Upstream transcription factor 1 (USF1) Ser309	Increase binding activity to the E-box of the *A20* gene promoter region, increasing *A20* transcription, a negative regulator of NF-κB	[63]
	PTEN Tyr 336	Activates Akt/mTOR signaling	[30]
	TIM (Timeless) Ser586/ Thr991	Promotes its nuclear accumulation	[64]
	TRPM7	Regulate magnesium homeostasis and reprogram cellular bioenergetics	[65–67]
	SRC	Directly interacts with SRC and inhibits SRC basal activation independently of its phosphatase activity	[68]
**PRL2**	E3 ubiquitin ligase CBL Tyr 371	Decreased KIT ubiquitination and enhanced AKT and ERK signaling	[57]
	VCP/p97 Tyr805	Lysophagy via collaboration with specific cofactors in the ELDR complex	[55]
	CNNM3	Magnesium homeostasis	[69]
	TRPM7	Regulate magnesium homeostasis and reprogram cellular bioenergetics	[65–67]
	PTEN Tyr336	Augmenting NEDD4-mediated PTEN ubiquitination and proteasomal degradation	[30]
	beta-subunit of Rab geranylgeranyltransferase II (betaGGT II)	Prenyltransferase known to exclusively prenylate Rab proteins	[45]
	Roundabout guidance receptor 1 (ROBO1)	Axon guidance receptor and involved in lysosomal biogenesis	[70]
	AMP-activated protein kinase α2 (AMPKα2) Thr172	An energy sensor protein kinase that plays a key role in regulating cellular energy metabolism and function in mitochondrial integrity	[71]
	p53 Ser392	Dephosphorylation at serine 392 decreases p53 stability and activity, consequently, promote leukemia initiating cell proliferation and survival	[58]
**PRL3**	α-tubulin	Cellular mitosis	[61]
	CNNM4	Magnesium homeostasis	[72]
	PTEN Tyr336	Activates Akt/mTOR signaling	[30]
	Ezrin Thr567	Cytoskeletal rearrangement and metastatic progression	[73,74]
	Integrin α1	Enhance the phosphorylation and activation of ERK1/2	[75]
	Integrin β1	Enhance the phosphorylation and activation of ERK1/2	[76,77]
	SRC	Rho activation	[78]
	SHP-2	Blocking the gp130 (Tyr759)-mediated repression of STAT3 activity	[79]
	p38 MAPK	Resistance to apoptosis under microenvironmental stress, including UV, H_2_O_2_, and hypoxia	[80]
	Stathmin	Microtubule destabilization and motility	[81]
	CRMP2	Regulating cellular morphology	[82]
	Ubiquitin-specific protease 4 (USP4)	Regulates the growth, invasion, and metastasis in cancer models	[83]
	CNNM3	Magnesium homeostasis	[84]
	MMP14	Regulation of cancer cell migration and invasiveness	[85]
	ARF6	Promoting cell migration	[86]
	Golgi protein TMED10	A protein channel for vesicle entry and secretion	[87]
	TRF2	RNA-binding activity	[88]
	RAP1	Adaptor protein in shelterin complex mediating both protein–protein and protein–DNA interactions	[88,89]
	Rag GTPases	Activate mTORC1, which plays a central role in cell growth and metabolism	[90]
	mTOR1	Promote cell growth and metabolism	[91]
	FZR1 (CDH1)	A subunit of the anaphase-promoting complex (APC), it plays a crucial role in cell cycle progression by acting as a ubiquitin ligase that targets specific proteins for degradation.	[92]
	CDH22	A transmembrane glycoprotein involved in cell–cell adhesion and metastasis	[54]
	Aurora kinase A (AURKA)	A serine/threonine kinase that plays a crucial role in cell division, during mitosis and spindle formation	[92]
	β3-tubulin	Cytoskeleton reorganization and cell shape formation during migration	[93]
	Focal Adhesion Kinase (FAK) Tyr397	Migration of adherent cells	[76]
	Na^+^/H^+^ exchanger regulating factor 1 (NHERF1) pSer	Promote the translocation of dephosphorylated NHERF1 from the nucleus to the cytoplasm	[94]
	Elongation factor 2 (EF2)	To activate EF2 to start the translation process	[74]
	Phosphatidylinositol(4,5)bisphosphate	Involved in promoting cell motility	[40]
	Keratin 8 Ser73 and Ser431	Co-localization at cellular lamellipodia and ruffles	[95]
	LCK	T cell receptor signaling	[96,97]
	JAM-2	Junctional adhesion molecule that stabilizes and recruits JAM-3 in the junction complex on the cell–cell contacts through heterophillic interaction	[98]
	KIAA1199	Activation of the FGFR4/Wnt/β-catenin and EGFR signaling pathways	[99]
	CD3	T cell receptor signaling	[97]
	Nucleolin	Synthesis and maturation of ribosomes	[100]

To understand the role of PRLs in normal physiology and tumorigenesis, many research groups, including our laboratory, have developed transgenic and knockout models to investigate their *in vivo* functions at organismic levels. Studies in *C. elegans* showed that PRL deletion caused abnormal trafficking of lysosomal membrane [101]. PRL loss in *Drosophila* resulted in neural dysfunction and retinal degeneration [64,102–104]. On the other hand, overexpression of PRL in *Drosophila* led to growth suppression by inhibiting SRC [46]. Moreover, overexpression of PRL promoted ectopic axonal protrusions and synapses in *Drosophila* [105] and induced dose-dependent wing vein over-proliferation through modulation of TGF-β signaling [105].

Genetic manipulation of PRLs in mice has yielded important insights into their biological roles, revealing substantial functional redundancy and strong context dependency (Table 2). Findings from organismal models further show that PRL family members regulate diverse developmental, metabolic, and tumor-relevant processes *in vivo*, while also highlighting strong context dependence and partial functional redundancy across the family (Table 2).

**Table 2:** Summary of PRL function in physiology and disease in organismal models

Protein	Condition	Phenotype
**PRL1**	Deletion	- Impaired spermatogenesis in PRL2^+/−^ mice due to increased PTEN abundance [106]. - Protected mice from dermal fibrosis through inhibiting TGFβ signaling [68]. - Aggravated glucose homeostasis and hepatosteatosis in mice fed a high-fat diet [107]. - Endothelial deletion (Tie2 Cre) in mice increases cell adhesion molecule expression and exacerbates diet-induced atherosclerosis [63]. - Hepatic-deletion (Alfp Cre) is dispensable for normal liver development, postnatal growth, and hepatocyte differentiation, but results in a delayed onset of DNA synthesis following 70% partial hepatectomy due to a significant reduction in the phosphorylation of AKT and STAT3, and decreased cyclin E, cyclin A2, cyclin B1, and CDK1, although ultimate liver mass restoration remains unaffected [108].
	Overexpression	- BM-MSCs-specific manipulation improved the cholestatic liver disease phenotype in BDL rats [109]. - Endothelial-specific (Tie2 Cre) overexpressing mice inhibited vascular inflammation [63].
**PRL2**	Deletion	- Placenta insufficiency and growth retardation in mice due to increased PTEN abundance and reduced AKT signaling [110]. - Impaired spermatogenesis in mice due to increased PTEN abundance and impaired AKT signaling [106] - Insufficient HSC self-renewal in mice due to increased PTEN and impaired SCF/Kit signaling [111]. - Inhibits PTEN heterozygosity-induced lymphomas by elevating PTEN level and attenuating AKT activity [30]. - Increased PTEN and reduction in AKT pathway activation in an acute myeloid leukemia mouse model [112]. - Elevated PTEN with reduced AKT signaling in sarcomas and lymphomas arising in *Tp53*-deficient mouse models [113]. - Reduce the burden of FLT3-internal tandem duplications–driven leukemia and extend the survival of leukemic mice [56]. - Delay the development of KIT/D814V-driven myeloproliferative neoplasms in mice [57]. - Protect mice from dermal fibrosis through inhibiting TGFβ signaling [68]. - Animals displayed sex-dependent alterations in body composition, thermogenesis, and circadian energy metabolism [114]. - Endothelial-specific deletion of PRL2 in mice exhibits defective retinal vascular outgrowth, arteriovenous differentiation, and sprouting angiogenesis [115]. - Deficiency in myeloid cells promotes the development of severe malaria with lung injury [116]. - Deficiency in myeloid cells led mice to develop more severe passive systemic anaphylaxis with enhanced FcεRI-mediated mast cell activation [117]. - Deficiency in myeloid cells decreased bone mass and enhanced the number of differentiated osteoclasts in mice [118]. - Improved survival of PRL2-deficient myeloid cells after *in vivo* X-ray irradiation [119]. - *PRL2* loss enhances phagocyte activation, ROS production, Rac activity, and bacterial clearance, conferring resistance to lethal *Listeria* infection in mice [120]. - Increased rate of uncoupled respiration and lower intracellular Mg^2+^ [114]. - Serum magnesium levels were significantly elevated but not urine magnesium levels [21].
	Overexpression	- MMTV-PRL2 transgenic mice with mammary-specific overexpression of PRL2 did not develop spontaneous tumors; however, PRL2 overexpression accelerated mammary tumor formation in the presence of the MMTV-ErbB2 transgene [10]. - Prostate-specific overexpression of PRL2 drives spontaneous tumor initiation and development in mice due to reduction in PTEN abundance and AKT activation [121].
**PRL3**	Deletion	- Impaired AOM/DSS-induced tumor initiation and clonogenicity [122,123]. - Reduced tumor microvessel density and vascular permeability [124].
	Overexpression	- Disrupted intestinal epithelial homeostasis and intestinal stem cell fitness [125]. - A high dose of PRL3 in homozygous expression led to mice dying early [125].

As summarized in Table 2, genetic and organismal studies indicate that alteration of PRL expression does not uniformly lead to transformation across all tissues, but instead exert context-dependent effects on development, growth, survival, metabolism, regeneration, and tumorigenesis. This context dependence is important for interpreting the PRL–PTEN axis, as it suggests that the biological consequences of PRL-mediated PTEN suppression are shaped by tissue type, developmental state, and cooperating signaling networks.

Among the three family members, PRL2 knockout mice display more pronounced systemic phenotypes, including fetal-onset growth retardation, placenta insufficiency, splenic and testicular hypoplasia, and defects in spermatogenesis driven by increased apoptosis of kit-positive germ cells [21,110,114,126]. Many of these phenotypes overlap with those reported in *Akt1*-deficient mice, which display fetal growth retardation and impaired placental development [127–129], as well as with PTEN transgenic mouse models that exhibit reduced AKT signaling and growth suppression [130–132]. Consistent with these observations, deletion of PRL2 attenuates AKT signaling, at least in part through increased PTEN protein levels [30,49], further supporting functional convergence between PRL2 and the AKT–PTEN signaling axis. Subsequent studies established PRL2 as a critical regulator of hematopoietic stem/progenitor cell proliferation and T-cell differentiation [126], with multiple models converging on attenuated SCF/Kit-mediated PI3K/AKT signaling and elevated PTEN expression in the absence of PRL2 [126]. Beyond cell growth and survival, PRL2 knockout mice exhibit sex-dependent alterations in body composition, thermogenesis, and circadian energy metabolism, alongside disrupted magnesium homeostasis due to impaired PRL2–CNNM interactions, linking PRL2 to systemic metabolic control [21,114].

In contrast, PRL1 loss produces a milder phenotype, with PRL1 knockout mice developing largely normally aside from a modest increase in preweaning lethality [106]. This subtle phenotype is explained by its functional redundancy with PRL2, as PRL2 knockout males exhibit mild reproductive defects [106], while combined loss of PRL2 and a single PRL1 allele results in a further increase in PTEN and complete male infertility, and PRL1/PRL2 double knockout embryos fail to survive beyond E9.5, demonstrating their compensatory roles during early embryogenesis and spermatogenesis [106]. Conditional deletion of PRL1 in the liver further showed that PRL1 is dispensable for steady-state tissue homeostasis but required for proper cell-cycle timing during liver regeneration, although compensatory expression of other PRLs was not assessed [108].

Two independent PRL3 conditional knockout models have also been reported, but with divergent phenotypes: one study observed no overt metabolic abnormalities [133], whereas another identified male-specific reduction in body weight, body mass index, and increased postnatal lethality [123]. Although the molecular basis of these discrepancies remains unresolved, the recurring emergence of sex-dependent phenotypes across PRL2 and PRL3 models highlights an underappreciated role for PRLs in sexually dimorphic metabolic regulation.

Accumulating evidence demonstrates that overexpression of PRL1, PRL2, or PRL3 significantly enhances tumor growth across xenograft and syngeneic orthotopic mouse models [10,134–136]. For example, overexpression of PRL2 did not induce tumor formation but significantly accelerated ErbB2-driven tumorigenesis in a syngeneic orthotopic xenograft mouse model [10]. PRL3 deficiency reduces tumor burden in inflammation-driven colorectal cancer models [123]. However, overexpression studies have yielded context-dependent results: PRL3 overexpression fails to initiate tumorigenesis in the intestinal epithelium [125], and endothelial-specific overexpression of PRL1 suppresses vascular inflammation without inducing malignant transformation [63]. Together, these findings suggest that the oncogenic activity of PRLs is highly dependent on tissue context and cooperating oncogenic signals. Recent studies in our lab using genetically engineered mouse models further suggest that PRL2, the most abundantly expressed PRL family member in mammalian tissues, may possess broader oncogenic potential. Notably, systemic overexpression of PRL2 has been found to drive spontaneous tumorigenesis in multiple tissues and reduces survival in mice, supporting a role for PRL2 as a driver of tumorigenesis *in vivo* [121].

Collectively, gene knockout studies have revealed that PRLs are largely dispensable for basal tissue maintenance but become critical under conditions of developmental stress, regeneration, metabolic demand, or lineage-specific signaling. These models therefore provide a powerful framework for interrogating PRL function in context-dependent processes, particularly in stem cell regulation, metabolic adaptation, and oncogenic signaling. Future studies should combine them with tissue-specific deletions, disease models, and pathway-focused perturbations, rather than relying solely on whole-body knockout phenotypes. Among the downstream pathways influenced by PRLs, PTEN is particularly important because it links PRL activity to a dosage-sensitive tumor suppressor axis with broad consequences for cell survival, proliferation, and tumor progression.

## PTEN as a dosage-sensitive tumor suppressor

PTEN was independently identified in 1997 by two groups investigating glioblastoma and prostate cancer cell lines, mapping the tumor suppressor gene to chromosome 10q23 [137,138]. PTEN is a well-established tumor suppressor with dual lipid and protein phosphatase activities [137]. Functionally, PTEN antagonizes the PI3K pathway through dephosphorylating PIP_3_ at the 3-position [139,140]. The conversion of PIP_3_ to PIP_2_ limits AKT activation and restrains multiple pathways, including proliferation [139,140], apoptosis [139,140], angiogenesis [141], and cell size [142].

Unlike classical tumor suppressors conforming to the classical two-hit paradigm of tumor suppressor inactivation, PTEN is haploinsufficient, such that even partial loss of its expression or activity is sufficient to promote tumor susceptibility [143–145]. This principle is supported by spontaneous tumor development in *Pten* heterozygous mice [143,146–148] and by frequent reduction of PTEN expression in human cancers, such as glioma, prostate, mammary, colon, and lung cancers [143], even in the absence of *PTEN* mutation or deletion [143,149]. Indeed, decreased PTEN expression has been documented in 70% of surgically isolated prostate tumors, underscoring the importance of PTEN dosage in cancer suppression [150,151]. Subsequent investigations revealed that monoallelic alterations at the *PTEN* locus are highly prevalent across multiple sporadic malignancies, occurring in approximately 50%–80% of sporadic endometrial carcinomas and 30-50% of breast, colon, and lung cancers [138,152]. Germline *PTEN* mutations also underlie a spectrum of autosomal dominant syndromes characterized by developmental abnormalities, neurological manifestations, multiple hamartomas, and elevated susceptibility to breast, thyroid, and endometrial cancers [153,154].

Given the strong cancer susceptibility to variations in PTEN abundance, PTEN is subject to extensive and delicate post-translational and post-transcriptional regulations. PTEN undergoes extensive post-translational modification, including phosphorylation, sumoylation, oxidation, acetylation, methylation, ribosylation, neddylation, and ubiquitination at multiple sites, many of which are essential for its phosphatase activity, stability, nuclear localization, and interactions with other molecules [155–161]. The C-terminal phosphorylation at Ser380/Thr382/Thr383 residues cluster enhances PTEN stability [162]. On the other hand, phosphorylation at Tyr336 by the SRC family kinase RAK protects PTEN from NEDD4-1-mediated polyubiquitination and degradation [163]. This is particularly important because loss of RAK reduces PTEN stability, enhances breast cancer cell proliferation, invasion, transformation, and tumor growth, identifying RAK as an upstream tumor-suppressive regulator of PTEN protein homeostasis [163].

PTEN is also subject to post-transcriptional repression by microRNAs, most notably miR-21, one of the most consistently upregulated oncogenic miRNAs across cancers. In colorectal, hepatocellular, and cervical cancers, miR-21 inversely correlates with PTEN expression and promotes proliferation, survival, migration, and invasion, whereas PTEN restoration or miR-21 inhibition counteracts these phenotypes [164–166]. In hepatocellular carcinoma, PTEN was further identified as a direct target of miR-21, linking its repression to focal adhesion kinase activation and upregulation of MMP-2 and MMP-9 [165]. Accordingly, the miR-21/PTEN/PI3K–AKT axis is now recognized as a recurrent mechanism of tumor progression and a potential therapeutic target [167]. miR-21 can also be induced by microenvironmental cues, as shown in chronic lymphocytic leukemia, where ZAP-70-dependent signaling increases miR-21 expression and promotes leukemic cell survival by suppressing tumor suppressor targets [168].

## PRLs as negative regulators of PTEN

**Figure 1:**
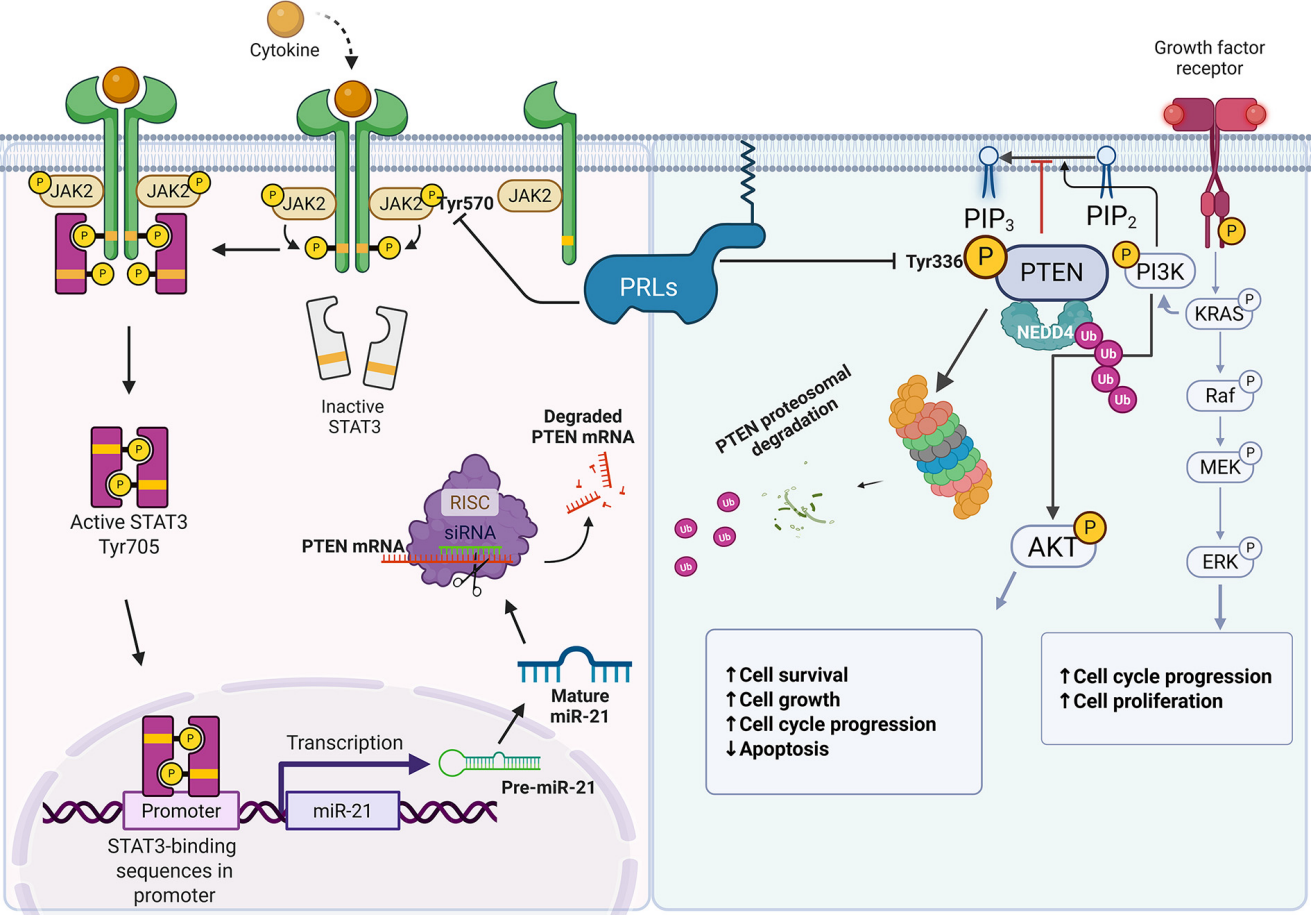
Mechanisms of PTEN regulation by the PRL phosphatases Multilevel regulation of PTEN by PRL phosphatases via post-transcriptional and post-translational mechanisms to amplify oncogenic signaling. PRLs promote oncogenic signaling through coordinated suppression of the tumor suppressor PTEN via two different regulatory mechanisms. On the right, PRLs regulate PTEN post-translationally through modulation of PTEN phosphorylation at Tyr336. Dephosphorylation of this residue promotes recruitment of the E3 ubiquitin ligase NEDD4, leading to PTEN ubiquitination and proteasomal degradation, further lowering PTEN protein levels. The reduction in PTEN relieves inhibition of the PI3K–AKT signaling axis, resulting in increased PIP_3_ accumulation and activation of downstream signaling pathways. Activated AKT enhances anabolic processes. On the left, PRLs can also regulate PTEN post-transcriptionally through activation of the JAK2–STAT3 signaling pathway by dephosphorylation of JAK2 Tyr570, leading to phosphorylation of STAT3 at Tyr705 and its subsequent nuclear translocation. Activated STAT3 binds to regulatory elements in the miR-21 promoter, enhancing transcription of primary miR-21 transcripts. After processing into mature miR-21, the microRNA is incorporated into the RNA-induced silencing complex, where it binds PTEN mRNA and promotes post-transcriptional repression and degradation, thereby reducing PTEN expression. Created in BioRender. AbouShanab, A. (2026) https://BioRender.com/iywjvlp

Among the negative regulatory pathways suppressed by PRLs, PTEN has emerged as the most compelling mechanistic link between PRL activity and sustained PI3K–AKT signaling. We uncovered two distinct biochemical mechanisms by which PRL2 downregulates PTEN at both post-transcriptional and post-translational levels (Figure 1). We found that PTEN is a putative PRL2 substrate, and that PRL2 downregulates PTEN by dephosphorylating Tyr336 residue of PTEN [30]. This dephosphorylated form of PTEN is ubiquitinated by NEDD4-1-mediated polyubiquitination and proteasomal degradation [30]. We also found that the mechanism may apply to all PRLs since PRL1 and PRL3 can also promote PTEN polyubiquitination and degradation by catalyzing PTEN Tyr336 dephosphorylation [30]. The capacity of PRL2 to reduce PTEN level establishes a mechanistic and biochemical framework that explains its oncogenic potential.

While NEDD4 has been identified as a critical E3 ubiquitin ligase targeting PTEN for destruction [160], its universal requirement remains a subject of ongoing debate, as some genetic deletion models show variable impacts on baseline PTEN stability across different tissues [169,170]. However, these physiological discrepancies do not invalidate individual regulatory pathways; instead, they highlight that the downstream routes of the PRL–PTEN axis are highly context-dependent. Indeed, direct comparative studies evaluating candidate E3 ligases (including NEDD4, WWP2, and XIAP) demonstrate that expression of NEDD4, but not XIAP or WWP2, dramatically elevates PTEN polyubiquitination in cooperation with active PRLs [30]. Furthermore, CRISPR–Cas9-mediated deletion of NEDD4 has been shown to largely abolish PRL-induced PTEN ubiquitination, successfully doubling the half-life of the PTEN protein [30]. This strong functional dependency underscores that PRLs can leverage alternative mechanisms to suppress PTEN in other cellular backgrounds.

Recently, we also showed that miR-21 expression can be upregulated by all PRLs and positively correlated with their expression across multiple tumor types [17]. miR-21 is known to control PTEN levels by directly targeting the 3′UTR of PTEN mRNA, causing its degradation [17]. Moreover, we uncovered that PRL2 dephosphorylates JAK2 at Tyr570, an inhibitory phosphorylation site, thereby activating STAT3 phosphorylation by JAK2 and downstream transcription. This STAT3-mediated upregulation of miR-21 leads to decreased PTEN expression [17]. This PRL2-mediated post-transcriptional control of PTEN was confirmed in breast cancer cells, where PRL2 upregulation promoted STAT3 expression and miR-21 expression and downregulated PTEN mRNA [17].

Accordingly, pharmacologic or genetic inhibition of PRL2 represents a rational therapeutic approach to reinstate PTEN function and counteract malignancies driven by PTEN insufficiency. Consistent with this concept, genetic ablation of PRL2 was shown to restore PTEN expression and significantly enhance overall survival in models of PTEN-haploinsufficiency-associated acute myeloid leukemia and related tumor systems [112]. In addition, we also showed that PRL2 loss or pharmacological inhibition in *Tp53*-deficient mice attenuates tumor growth through upregulating PTEN protein abundance and attenuating AKT activity [113]. Thus, augmenting PTEN function represents a promising therapeutic strategy in cancer treatment. The efficacy of several targeted therapies, particularly PTK inhibitors, is significantly improved when tumors retain functional PTEN [171–173].

Consistent with this, adenoviral-mediated reintroduction of PTEN has been shown to markedly suppress tumor growth in multiple animal models [174–176], highlighting the potential of PTEN restoration as an anticancer approach. Importantly, increasing PTEN levels appears to be well tolerated: overexpression of PTEN in cells that already express physiological levels produce minimal adverse effects [177], and mice with systemic PTEN elevation remain healthy while exhibiting enhanced resistance to tumor development [130]. Despite the potential promise of PTEN restoration, the physiological control of PTEN expression and proteasomal degradation remains of importance to unravel upstream regulators for designing efficacious therapeutics to target PTEN dependencies in cancer.

Additionally, our studies using genetically engineered mouse models demonstrate that PRL2 overexpression drives prostate cancer initiation and progression, particularly in aged animals, providing the *in vivo* support for its oncogenic function. Mechanistically, PRL2-driven tumorigenesis is associated with downregulation of the tumor suppressor PTEN, leading to activation of PI3K/AKT signaling [121]. Collectively, these findings support PRLs as negative regulators of PTEN and suggest that PRLs may function as oncogenic drivers by enhancing AKT signaling through downregulation of PTEN.

## Open questions and future directions

Despite growing evidence that PRLs can post-transcriptionally and post-translationally downregulate PTEN, several fundamental questions remain unresolved. (1) Are JAK2 and PTEN universal physiological substrates of all PRLs? Although biochemical studies demonstrate that PRL2, and possibly PRL1 and PRL3, can suppress PTEN expression through JAK2 Tyr570 and PTEN Tyr336 dephosphorylation, it remains unclear whether these mechanisms operate broadly across tissues and tumor types. Genetic redundancy among PRL family members complicates the interpretation of knockout models. Tissue-specific deletions are required to determine whether PTEN regulation is a conserved function or a context-dependent phenomenon. (2) Do PRLs function primarily as phosphatases or signaling scaffolds? Many PRL-dependent phenotypes require catalytic activity and membrane localization, yet some biological effects may arise from protein–protein interactions, such as PRL–CNNM binding. Distinguishing enzymatic substrate turnover from scaffold-mediated signaling remains essential for understanding PRL biology and for designing selective inhibitors. (3) In physiological systems, how is the PRL–PTEN axis regulated? (4) Do metabolic stress or magnesium flux influence PRL mediated PTEN regulation? (5) Can PRL inhibition restore PTEN function therapeutically? Work from preclinical model suggests that PRL2 inhibition stabilizes PTEN and suppresses tumor growth; however, several issues remain unresolved, including the selectivity of PRL inhibitors and the potential toxicity associated with PRL’s roles in metabolism and development. (6) Are the oncogenic functions of PRLs driven solely by PTEN suppression, or do additional PRL substrates make parallel contributions? In addition to serving as negative regulators of PTEN [30,113], PRLs have been reported to dephosphorylate VCP/p97 at Tyr805 to promote lysophagy [55] and to enhance receptor tyrosine kinase signaling through dephosphorylation of the E3 ubiquitin ligase CBL at Tyr371 [56,57]. These findings suggest that PRLs promote tumorigenesis through multiple substrate-specific pathways that converge on survival, proliferation, and proteostasis, depending on cancer type dependencies. Defining the relative contribution of JAK2-, PTEN-, VCP/p97-, and CBL-dependent mechanisms in different tumor contexts will be essential for understanding PRL biology and for predicting the therapeutic consequences of PRL inhibition.

## Conclusions

PRL phosphatases have emerged as important drivers of oncogenic signaling in part by suppressing the tumor suppressor PTEN. Genetic, biochemical, and *in vivo* studies demonstrate that PRLs enhance PI3K–AKT signaling by lowering PTEN levels through both post-translational and post-transcriptional mechanisms. These findings provide a unifying framework for understanding the pleiotropic oncogenic phenotypes associated with PRL overexpression. However, key aspects of PRL biology remain unresolved. The extent to which PTEN represents a major downstream target of PRLs, the relative contributions of PRL catalytic versus scaffold functions, and the degree of redundancy among PRL family members are still under investigation. Addressing these questions will require tissue-specific genetic models, endogenous-level biochemical studies, and the development of selective PRL inhibitors. Given the central role of PTEN haploinsufficiency in tumorigenesis, targeting PRLs represents a promising strategy to restore PTEN function and suppress cancer progression. Continued integration of structural biology, *in vivo* genetics, and translational research will be essential to determine whether modulation of the PRL–PTEN axis can be exploited therapeutically.

## Perspectives

- PRL phosphatases act as upstream dismantlers of tumor-suppressive signaling, with PTEN emerging as a central mechanistic target. PTEN stands out as a particularly important node because it is a major antagonist of PI3K–AKT signaling and a dosage-sensitive tumor suppressor whose deficiency has broad consequences for cancer progression.
- Through coordinated post-translational and post-transcriptional suppression of PTEN, PRLs amplify PI3K–AKT signaling and help explain the broad oncogenic phenotypes associated with PRL overexpression. At the protein level, PRLs promote PTEN destabilization through Tyr336 dephosphorylation and NEDD4-mediated ubiquitination, while at the transcript level, they reduce PTEN expression through the upregulation of the JAK2–STAT3–miR-21 axis.
- This integrated view identifies the PRL–PTEN axis as both a conceptual framework for understanding PRL biology and a promising avenue for therapeutic intervention. Because PTEN is haploinsufficient, even partial PRL-mediated reductions in PTEN abundance may be sufficient to drive meaningful biological effects in tumorigenesis. Accordingly, targeting PRLs may offer a strategy not only to inhibit the oncogenic phosphatase activity, but also to restore tumor-suppressive buffering in PTEN-insufficient cancers.

## Open Access

Open access for this article was enabled by the participation of Purdue University in an all-inclusive Read & Publish agreement with Portland Press and the Biochemical Society under an agreement with the Big Ten Alliance.

## Abbreviations

CNNM: magnesium transporters of the cyclin M
PI3K: phosphatidylinositol 3-kinase
AOM/DSS: azoxymethane/dextran sodium sulfate
CSK: C-terminal SRC kinase
PI3K: phosphatidylinositol 3-kinase
PRL: phosphatase of regenerating liver
PTEN: phosphatase and tensin homolog deleted on chromosome 10
PTK: protein tyrosine kinase
PTP: protein tyrosine phosphatase
TGFβ: transforming growth factor beta
HSC: hematopoietic stem cell
FGFR: fibroblast growth factor receptor
EGFR: epidermal growth factor receptor
mTORC1: mammalian target of rapamycin complex 1
MMP: matrix metalloproteinase
TRPM7: transient receptor potential cation channel subfamily M member 7
SHP-2: Src homology 2 domain-containing phosphatase-2
CRMP2: collapsin response mediator protein 2
ARF6: ADP-ribosylation factor 6
RAP1: Ras-related protein 1
LCK: leukocyte C-terminal Src kinase
JAM2: junctional adhesion molecule 2
AKT: protein kinase B (PKB)
ERBB2: erb-b2 receptor tyrosine kinase (HER2)
PIP: phosphatidylinositol 4,5-bisphosphate
NEDD4: developmentally down-regulated 4
WWP2: WW domain containing E3 ubiquitin protein ligase 2
XIAP: X-linked inhibitor of apoptosis protein
CRISPR: clustered regularly interspaced short palindromic repeats
Cas9: CRISPR-associated protein 9
JAK2: janus kinase 2
STAT3: signal transducer and activator of transcription 3
MMTV: mouse mammary tumor virus
FcεRI: Fc fragment of IgE receptor I
ROS: reactive oxygen species
RAC1: RAS-related C3 botulinum toxin substrate 1
FLT3: Fms-like tyrosine kinase 3
SCF: stem cell factor
BDL: bile duct ligature
Alfp: alpha-fetoprotein
Tie2: tunica interna endothelial cell kinase (Tek)
Wnt: wingless-related integration site
